# A Perspective on the Potential Zoonotic Role of *Streptococcus agalactiae*: Searching for a Missing Link in Alternative Transmission Routes

**DOI:** 10.3389/fmicb.2018.00608

**Published:** 2018-03-28

**Authors:** Ana C. N. Botelho, Ana F. M. Ferreira, Sergio E. L. Fracalanzza, Lucia M. Teixeira, Tatiana C. A. Pinto

**Affiliations:** Instituto de Microbiologia Paulo de Goes, Universidade Federal do Rio de Janeiro, Rio de Janeiro, Brazil

**Keywords:** *Streptococcus agalactiae*, human, bovine, zoonotic transmission, antimicrobial resistance

*Streptococcus agalactiae*, or group B *Streptococcus* (GBS), emerged as a leading cause of human neonatal infections after seven decades of being exclusively associated with mastitis in dairy herds (McCracken, 1973; Keefe, 1997). Although currently available data suggest that GBS jumped from animals to humans in a certain moment of the evolution, becoming then fixed and specialized to the new host, it is still debatable if this zoonotic potential remains nowadays (Oliveira et al., 2006; Sørensen et al., 2010; da Cunha et al., 2015). In this opinion article, we highlight a hypothesis, based mainly on multilocus sequence typing (MLST)^1^ and antimicrobial resistance data, on routes through which *S. agalactiae* could cross the interspecies barrier and be transmitted between bovines and humans.

Bovine mastitis caused by GBS represents a huge economic problem for the dairy industry, especially because this microorganism is highly contagious within a farm and rapidly reduces milk productivity. During the last decades, certain developed countries have witnessed a drastic reduction of GBS occurrence due to implementation of programs specifically designed for the control of bovine mastitis. Among other aspects, such programs are based on the administration of antimicrobial infusions to the entire herd to treat and prevent the disease, and were successful since this microorganism remained susceptible to most of the antimicrobial agents of choice (Keefe, 1997). However, in most developing countries, implementation of mastitis control programs has been a slow and inadequate process, leading to a historic misuse of antibiotics in the veterinary field. In addition, the farming system in such countries usually includes a high number of small producers who may be unaware of appropriate protocols for controlling GBS mastitis. Among the top five milk producers worldwide, three are developing countries with no mastitis control guidelines currently established, including Brazil, China and India (FAO, http://www.fao.org/dairy-production-products/en/)^2^. Nevertheless, in Brazil for example, there are 180 antimicrobial formulations approved for treatment and prevention of bovine mastitis (http://www.sindan.org.br)^3^. Tetracyclines are present in more than thirty of the recommended formulations and macrolides in more than seven. According to the World Organisation for Animal Health (OIE), between 2010 and 2015, tetracyclines and macrolides were the two classes of antibiotics most commonly used in animals worldwide, representing 48 and 15%, respectively, of all antimicrobial agents (OIE, 2016).

*Streptococcus agalactiae* is also found colonizing the gastrointestinal and genitourinary tracts of 10–35% of human populations (Schrag and Verani, 2013). Asymptomatic colonization of pregnant women is the main source of neonatal contamination by GBS, and guidelines to prevent this transmission route were established by the US Centers for Disease Control and Prevention (2010). As for mastitis control programs, such guidelines rely on the administration of antibiotics to eradicate GBS colonization in the moment of labor, characterizing the intrapartum antibiotic prophylaxis (IAP). Again, developed countries experienced a drastic reduction in GBS neonatal infections after national implementation of IAP, especially because this microorganism remained mostly susceptible to the drugs of choice (Centers for Disease Control and Prevention, 2010). According to CDC guidelines, IAP should be done preferentially with penicillin or ampicillin. In the case of allergy to beta-lactams, erythromycin, clindamycin, or vancomycin represent alternatives (Centers for Disease Control and Prevention, 2010). In many developing countries, however, there are no national consensus or guidelines regarding such prophylactic approach, and adhesion to IAP is likely rare.

Recently, da Cunha et al. (2015) suggested that the emergence of GBS clones able to cause human infections in the 1960's was associated with the acquisition of tetracycline resistance determinants (especially the *tet*M gene). These authors have suggested that few GBS clones, originally adapted to the bovine host, acquired *tet*M genes via conjugative transposons of the Tn*916* family, which were then selected by the widespread use of tetracycline in human medicine during the 1950's. The clones that were also highly pathogenic to humans were fixed and rapidly spread throughout the world. Thus, tetracycline resistance mediated by *tet*M gene can be considered a marker of success among human-adapted GBS lineages but not necessarily it is among those of bovine origin. Indeed, very high ratios of tetracycline resistance, ranging from 70 to 95%, are usually observed in human GBS strains in different countries, among which *tet*M is harbored by up to 90% (Sharmila et al., 2011; Usein et al., 2012). In turn, bovine GBS isolates are usually less resistant to tetracycline, with percentages ranging from 15 to 60%, among which *tet*M can be found in only 2–20% (Dogan et al., 2005; Rato et al., 2013).

While acquisition of tetracycline resistance might have been essential for the initial emergence of GBS as a major agent of human infections, acquisition of additional resistance markers, such as erythromycin and clindamycin resistance, might have been an important step for its evolution as a human pathogen. In the 1990's, erythromycin-resistant GBS isolates emerged as a significant cause of infections among non-pregnant adults (Jackson et al., 1995; Amundson et al., 2005). In addition, the extensive application of CDC's IAP led to the emergence of erythromycin and clindamycin resistance also among GBS isolates recovered from pregnant women and neonates (Morales et al., 1999; Centers for Disease Control and Prevention, 2010). In fact, clindamycin-resistant *S. agalactiae* has been appointed by CDC as one of the current concerning antimicrobial resistance threats (Centers for Disease Control Prevention, 2013). Among GBS isolates, the most common genetic determinant for erythromycin resistance is *erm*B, which is frequently found with *tet*M gene in the same conjugative transposon, such as those of Tn*916* family (da Cunha et al., 2015; Flores et al., 2015). The proportion of infections associated with GBS isolates resistant to erythromycin and clindamycin has been increasing steadily since 2000, and nearly 50 and 30% of human GBS isolates in the United States show resistance to these drugs, respectively (Imperi et al., 2011; Back et al., 2012; Centers for Disease Control Prevention, 2013). On the other hand, this trend has not been observed among bovine GBS isolates in the USA (Lindeman et al., 2013).

In addition to differences in antimicrobial susceptibility profiles, *S. agalactiae* strains recovered from human and bovine sources usually belong to different and completely separate genetic lineages. According to MLST data, most human GBS isolates circulating worldwide are represented by only five clonal complexes, including CC1, CC10, CC17, CC19, and CC23; each one comprising a number of different sequence types (ST). In turn, most bovine isolates belong to CC67, a clonal complex exclusively detected among GBS strains of bovine origin (da Cunha et al., 2015). Human- and bovine-adapted lineages differ from each other in a number of characteristics, including the panel of virulence factors. For example, several studies highlight that GBS strains from different origins usually harbor different pili variants; while the pilus island type 2a (PI-2a) is more common in human strains, pilus island type 2b (PI-2b) is more frequent in bovine isolates (Martins et al., 2013; Otaguiri et al., 2013; Pang et al., 2017). Such a divergence is in agreement with the fact that the human and bovine organisms represent very different environments and, thus, can lead to different evolution paths. However, there is increasing evidence that, within dairy farms, GBS can survive in other places besides the bovine udder (Manning et al., 2010; Jorgensen et al., 2016). Such studies highlight the existence of two transmission cycles. The first and more common is a contagious transmission cycle occurring during the milking procedure, while the second and less common is an environmental transmission cycle sustained by fecal shedding and leakage of milk from infected udders. Apparently, the bacterium can survive in the gastrointestinal tract of animals, in milk fat, on bovine skin, on milkers' hands and clothes and in fresh water, and through such alternative routes could more easily get to colonize humans as well.

Moreover, these data underscore the concept that certain GBS strains, or lineages, might be more versatile than initially thought, representing a evolutionary link between human- and bovine-adapted clones. In our opinion, a candidate that suits this profile is CC103. This clonal complex seems to be a successful lineage in the alternative transmission route of GBS, being the most commonly found in GBS strains recovered from the farm environment in Norway (Jorgensen et al., 2016). In addition, CC103 has been reported among bovine isolates from Brazil, China, Denmark, Finland, Norway, Sweden and Thailand; and among human isolates from Brazil, China, Finland, Kenya and the Netherlands (Oliveira et al., 2006; Zadoks et al., 2011; Yang et al., 2013; Carvalho-Castro et al., 2017; https://pubmlst.org/sagalactiae/). In many of these countries, such as Denmark, CC103 has been detected only recently, being linked to the reemergence of GBS as a significant cause of bovine mastitis after many years of successful mastitis control (Zadoks et al., 2011).

However, it seems that in some places CC103 might be circulating for a longer time. In Brazil specifically, CC103 has been found among human and bovine isolates recovered in the 1980's (Oliveira et al., 2006). In addition, Brazil shows a peculiar scenario regarding percentages of tetracycline and erythromycin resistance among GBS isolates. While human *S. agalactiae* strains usually show high percentages of tetracycline resistance as in other countries, percentages of erythromycin resistance are generally very much lower, not surpassing 19% (d'Oliveira et al., 2003; Duarte et al., 2005; Corrêa et al., 2009; Pinto et al., 2013; Dutra et al., 2014). Even more unexpected are the ratios of tetracycline and erythromycin resistance among bovine GBS isolates. Since the 1980's, erythromycin resistance percentages are higher than 10% and tetracycline resistance is detected in 45–90% of bovine strains (Duarte et al., 2004, 2005; Pinto et al., 2013). In addition, antimicrobial-resistant bovine GBS isolates recovered in Brazil frequently harbor resistance determinants that represent successful markers of human lineages, such as *erm*B (present in up to 90% of the isolates) and *tet*M (found in up to 53% of the isolates) (Duarte et al., 2004; Pinto et al., 2013). Interestingly, CC103 has been linked to tetracycline and erythromycin resistance in different countries (Oliveira et al., 2006; Zadoks et al., 2011; Yang et al., 2013; Carvalho-Castro et al., 2017; https://pubmlst.org/sagalactiae/), suggesting that the peculiar panorama of antimicrobial resistance seen among bovine GBS isolates in Brazil might be due to the broad circulation of CC103.

Recently, the complete genome sequence of a ST103 GBS strain recovered from human oropharynx in Brazil was made available, and a comparative genomic analysis including other 21 GBS genomes representative of different origins and clonal complexes, revealed that this isolate was distantly related to the most common CC detected in human and bovine GBS strains, being more closely associated with CC7, a clonal complex comprising strains recovered from humans and fish and previously suggested to be of zoonotic origin (Liu et al., 2013; Björnsdóttir et al., 2016; de Aguiar et al., 2016). By MLST, CC7, and CC103 also show some degree of relationship, with ST314 (CC103) presenting five out of the seven MLST loci identical to ST255 (CC7) (https://pubmlst.org/sagalactiae/).

Regarding the panel of virulence factors, CC103 isolates usually resemble the profile detected in bovine GBS strains, being PI-2b the pilus variant most commonly found in this complex (Yang et al., 2013; Carvalho-Castro et al., 2017). However, it should be noted that certain highly virulent lineages to humans, such as CC17, harbor PI-2b instead of PI-2a (da Cunha et al., 2015), suggesting that different virulence profiles can be associated with successful infections in different hosts. Indeed, *in vitro* studies have indicated that ST103 can be pathogenic to humans (de Aguiar et al., 2016).

Thus, although a robust epidemiological database is not yet available for GBS isolates circulating in most developing countries, especially those placed among the top five milk producers worldwide, the peculiar panorama observed in some of them, such as Brazil, suggests that CC103 might have been circulating for a long time in these places, among both bovine and human hosts. This may be due, at least in part, to the absence of well-established guidelines for treating and controlling GBS infections in such countries, leading to a historic misuse of antibiotics in veterinary and human medicine, which might have been selecting these antimicrobial-resistant clones over time. Moreover, if CC103 is characteristically a GBS lineage of developing countries, its recent emergence among dairy herds in certain developed countries could be the result of imported animals, and may suggest the possible future emergence of this CC among human GBS strains as well. Either way, CC103 can be highlighted as one of the most versatile GBS lineages, being found among bovine, human and environmental strains of *S. agalactiae*, and representing one of the missing links between human- and bovine-adapted GBS clones, especially in the alternative transmission routes.

Figure 1 summarizes the hypothesis raised in this opinion article, showing the clonal complexes mostly associated with each host and transmission route of *S. agalactiae*, and the distribution of clonal complex CC103 worldwide.

**Figure 1:**
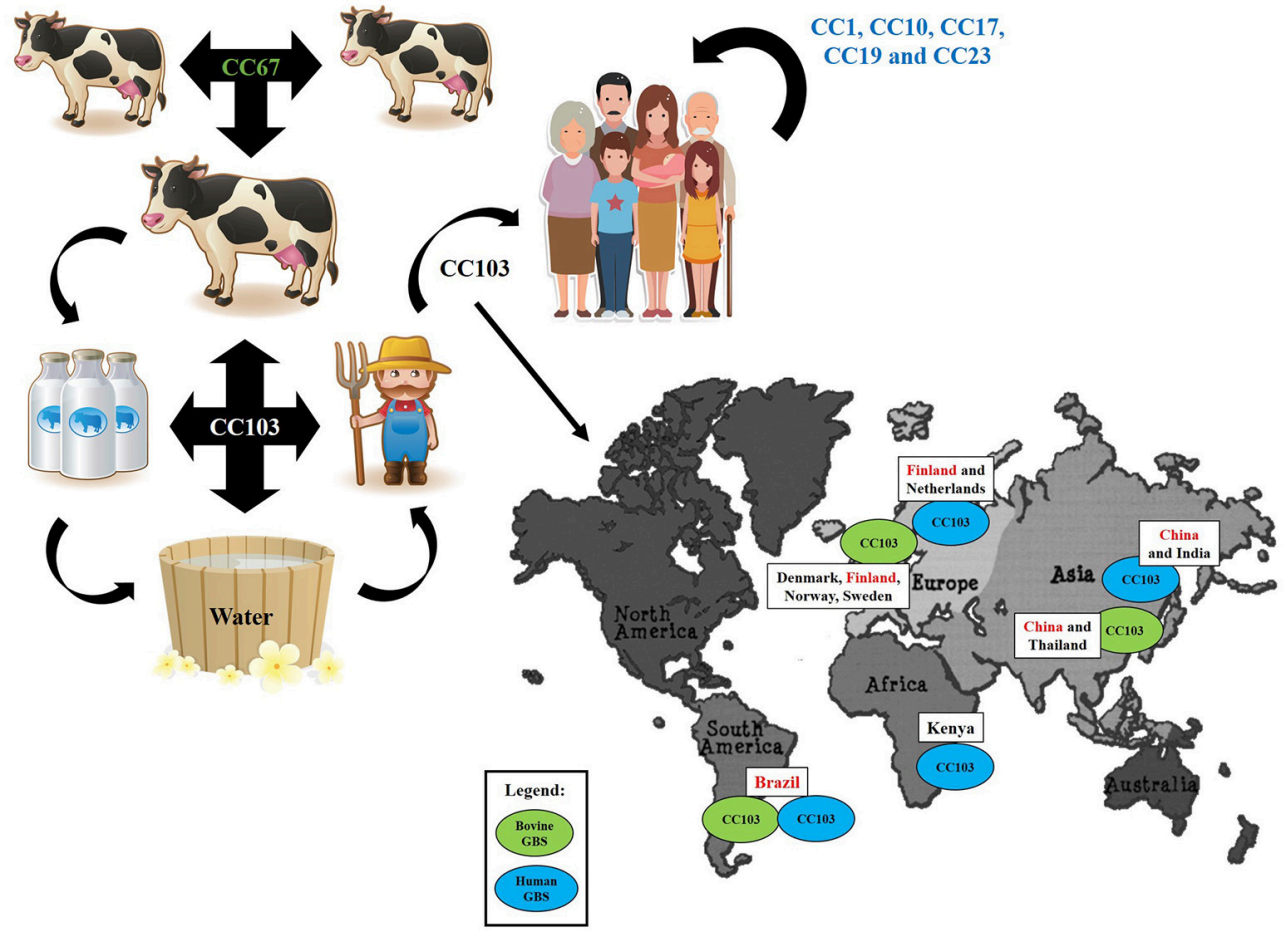
An illustrative summary of the hypothesis raised in this opinion article, showing the clonal complexes (CC) mostly associated with each host and transmission route of *Streptococcus agalactiae*, and the distribution of CC103, one of the missing links between human- and bovine-adapted clones in the alternative transmission routes, worldwide.

## Author contributions

AB and TP developed the concept of the manuscript and wrote the initial draft. AB, AF, SF, LT, and TP have critically read, advised on improvements concerning the science and general outline of the manuscript, approved the final version of the manuscript and agree with the opinions expressed here.

### Conflict of interest statement

The authors declare that the research was conducted in the absence of any commercial or financial relationships that could be construed as a potential conflict of interest.

## Footnotes

^1^MLST, Multilocus Sequence Typing. Available online at: https://pubmlst.org/sagalactiae/ (Accessed Nov 24, 2017).

^2^FAO, Food and Agriculture Organization of the United Nations. Available online at: http://www.fao.org/dairy-production-products/en/ (Accessed Nov 24, 2017)

^3^SINDAN, Sindicato Nacional da Indústria de Produtos Para Saúde Animal. Available online at: http://www.sindan.org.br (Accessed Nov 24, 2017)
